# Progressive Cranial Nerve Deficits in Granulomatosis With Polyangiitis: A Case Report

**DOI:** 10.7759/cureus.86364

**Published:** 2025-06-19

**Authors:** Ryan Waggoner, Imran Bitar, Manasa Pavuloori, Atheel Yako, Natasha Kizy

**Affiliations:** 1 School of Medicine, Oakland University William Beaumont School of Medicine, Rochester, USA; 2 Neurology, Corewell Health William Beaumont University Hospital, Royal Oak, USA

**Keywords:** anca-associated vasculitis, cranial nerve involvement, granulomatosis with polyangiitis (gpa), multidisciplinary diagnosis, neuroimmunology, systemic autoimmune disease

## Abstract

Granulomatosis with polyangiitis (GPA) is a rare systemic vasculitis that can involve multiple organ systems, including the nervous system. Central nervous system (CNS) involvement, especially in the form of cranial nerve neuropathy, is uncommon and may present with significant diagnostic challenges. This case report details the presentation, diagnostic workup, and management of a 60-year-old female who initially developed unilateral cranial nerve deficits, which progressed to bilateral involvement over an 11-day hospital course. The purpose of this case report is to highlight the importance of including GPA in the differential diagnosis in the setting of progressive cranial neuropathy, and to emphasize the need for a multidisciplinary approach for timely diagnosis and utilization of immunosuppressive treatment to minimize the chronic effects of the disease.

## Introduction

Granulomatosis with polyangiitis (GPA), formerly known as Wegener’s granulomatosis, is a rare, systemic necrotizing vasculitis that primarily affects small- to medium-sized vessels. This systemic vasculitis most commonly affects the upper and lower respiratory tracts, eyes, and kidneys [1]. Although GPA classically presents with pulmonary-renal syndromes and ENT manifestations in most scenarios, its clinical spectrum is diverse and can include central nervous system (CNS) involvement [2]. CNS manifestations may result from direct granulomatous inflammation, hypertrophic pachymeningitis, or vasculitis involving small cerebral vessels - potentially leading to ischemic stroke [3,4].

Neurological involvement is reported in approximately 15% of total GPA cases, but isolated cranial nerve involvement is a relatively rare phenomenon [5]. If not diagnosed and treated early, GPA-related neuropathies may lead to irreversible neurological deficits, including persistent facial palsy, ophthalmoplegia, or long-term visual impairment [4,6]. 

The etiology of GPA is thought to be multifactorial, involving genetic susceptibility, autoimmune dysregulation, and environmental exposures (e.g., infections or silica). It has an estimated incidence of 3 cases per 100,000 persons per year [1,7]. CNS symptoms may result from vasculitis of the vasa nervorum, direct granulomatous inflammation, or secondary effects from hypertrophic pachymeningitis [3]. Among cranial neuropathies, facial nerve palsy is occasionally reported, but bilateral or progressive deficits are uncommon and easily misdiagnosed [4].

Delayed diagnosis is documented in the literature when GPA presents with isolated neurological manifestations, often being mistaken for more common etiologies such as Bell’s palsy or Guillain-Barré syndrome variants [3-6]. In such cases, early recognition of GPA is crucial, as early immunosuppressive therapy can substantially improve outcomes and prevent irreversible neurologic damage [1].

The following case describes a patient with progressive cranial nerve deficits, initially diagnosed as Bell’s palsy, who was later confirmed to have GPA. The case emphasizes the importance of maintaining a broad differential when evaluating cranial neuropathies and underlines the role of prompt immunosuppressive therapy and multidisciplinary care in achieving desirable outcomes for affected patients.

## Case presentation

A 60-year-old non-Hispanic Caucasian female presented to the Emergency Department with right-sided facial drooping and an inability to close her right eye. Her past medical history included chronic sinusitis, hypertension, hyperlipidemia, and hypothyroidism. She was initially diagnosed with Bell’s palsy and treated with prednisone, 60 mg daily for seven days, and valacyclovir, 1000 mg three times daily for seven days. Despite initial treatment, her symptoms persisted, and over the course of several weeks, she developed worsening neurological complaints, including intermittent diplopia, progressive left-sided ptosis, and dizziness. Her facial droop began to worsen, and she noticed difficulty moving her left eye. Initial imaging at the time of presentation included a non-contrast head computed tomography (CT), which showed no acute intracranial hemorrhage, mass effect, or midline shift. A facial CT performed concurrently revealed paranasal sinus disease in the bilateral maxillary and ethmoidal sinuses; however, the facial nerve could not be adequately assessed by CT.

Approximately six weeks after symptom onset, she was admitted to the hospital for further evaluation. On admission, she reported severe fatigue, mild headache, and intermittent sinus pressure. Neurological examination revealed right-sided cranial nerve VII palsy, with complete facial weakness, inability to raise her eyebrow, and difficulty closing her right eye. Additionally, left-sided cranial nerve III deficits were noted, characterized by ptosis and ophthalmoplegia. No cognitive, motor, sensory, or cerebellar deficits were noted, and deep tendon reflexes were normal. The initial neuroimaging - including CT head, CT angiography of the head/neck, as well as magnetic resonance imaging (MRI) brain without contrast - was negative for any acute abnormality or enhancement. Symptoms were initially thought to be due to a neuromuscular disorder, specifically myasthenia gravis. However, the patient's extraocular deficits, including a left third nerve palsy with pupil-sparing, combined with preserved limb strength, normal reflexes, and the absence of bulbar symptoms, were atypical for myasthenia gravis. Serial neurologic exams demonstrated no fatigability, dysphagia, or dysarthria. This, in addition to a negative acetylcholine receptor (AChR) antibody panel, made myasthenia gravis less likely. Other causes, such as demyelinating neuropathies - including Miller Fisher syndrome - and demyelinating diseases like neurosarcoidosis, were also considered, given the evolving cranial nerve deficits. AChR antibodies were negative. She was initiated on pyridostigmine and prednisone, with consideration for possible seronegative myasthenia gravis, and was ultimately discharged with outpatient follow-up. During an outpatient ophthalmology appointment, she was noted to have an additional left cranial nerve VI deficit and was advised to return to the Emergency Department.

Throughout her hospitalization, her symptoms progressed further, and she developed additional cranial neuropathies. Ophthalmologic evaluation documented involvement of several cranial nerves, including left cranial nerves III, VI, and VII, in addition to the original right cranial nerve VII deficit. The patient reported progressive diplopia, facial drooping, and eyelid lag, with examination showing extraocular movement limitation and mild improvement with ice-pack testing. Although direct clinical documentation of cranial nerve V and IX symptoms was limited, mild facial numbness and oropharyngeal discomfort were intermittently noted during hospitalization, suggesting possible involvement of these nerves. She was evaluated by ophthalmology, neurology, infectious disease, and rheumatology specialists. Given her history of chronic sinus infections and progressive cranial neuropathies, vasculitis was considered a primary differential diagnosis.

Imaging and laboratory findings

**Figure 1:**
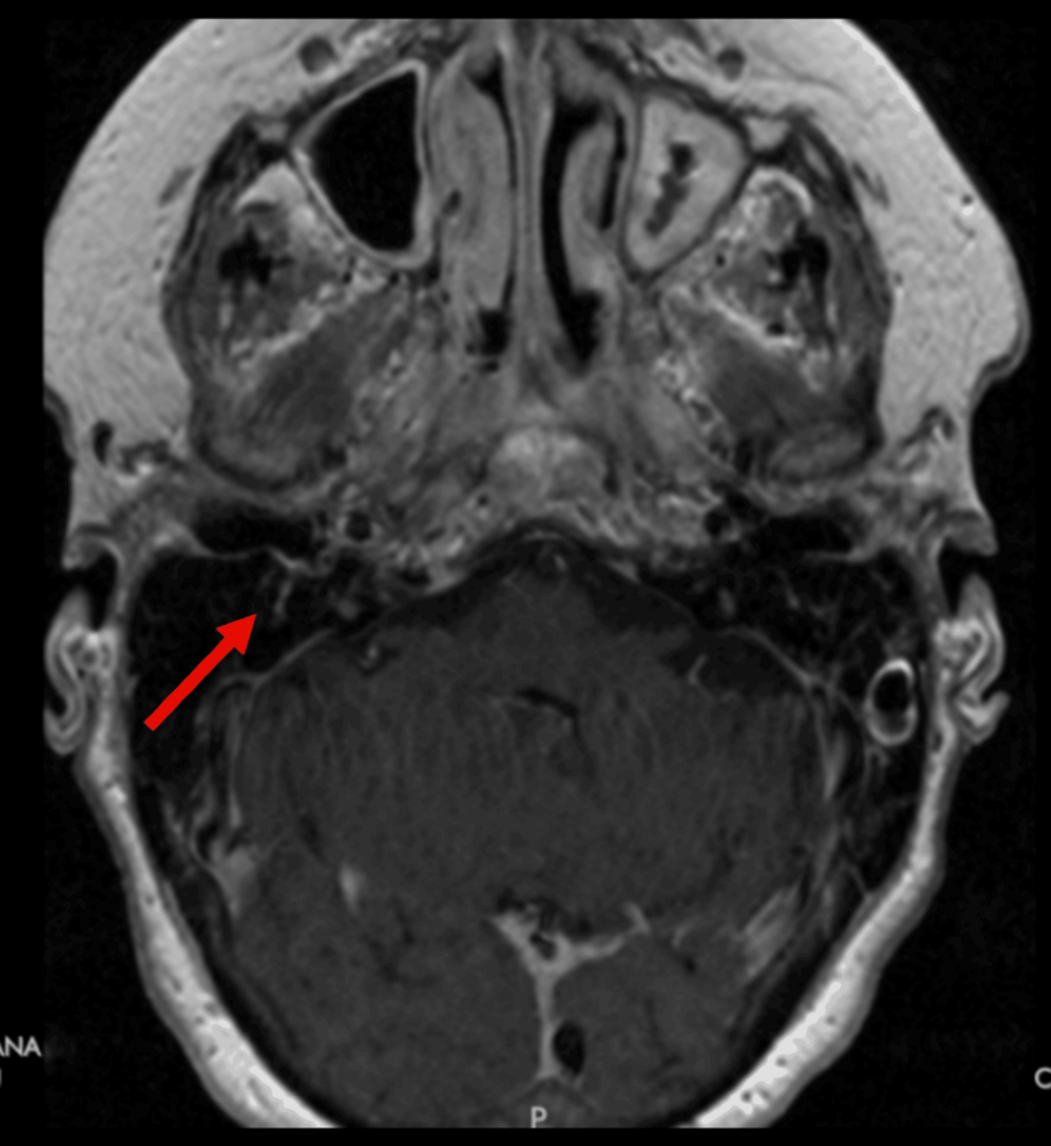
Axial post-contrast MRI of the brain demonstrating enhancement of the right cranial nerve VII (facial nerve) (arrow), consistent with an inflammatory process. No corresponding enhancement is noted on the left. MRI, Magnetic Resonance Imaging

**Figure 2:**
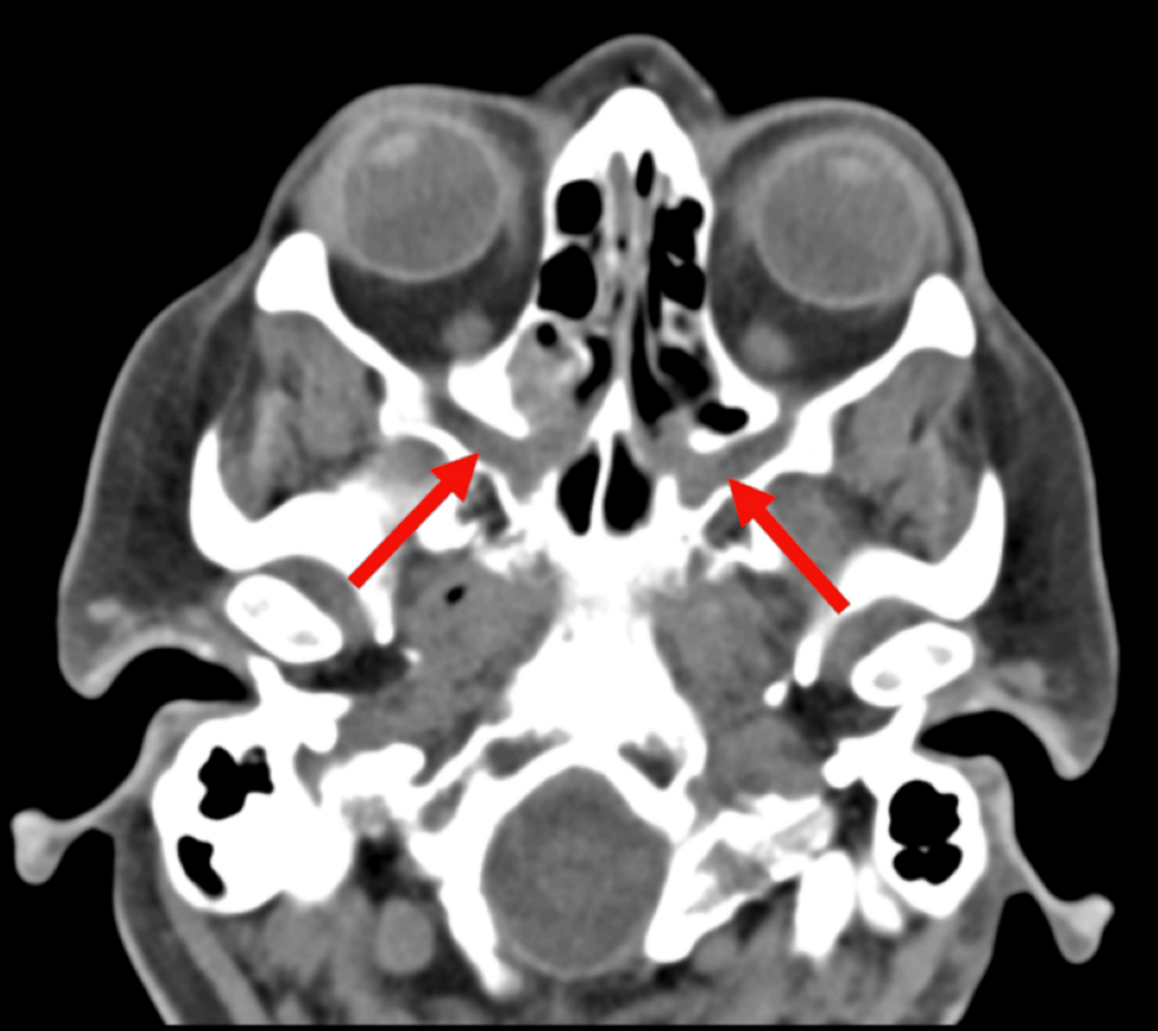
Axial head CT with IV contrast demonstrating abnormal soft tissue thickening in the pterygopalatine fossa (arrows), extending into the sphenopalatine foramen and involving the pterygoid canal, concerning for an infiltrative process. CT, Computed Tomography

**Figure 3:**
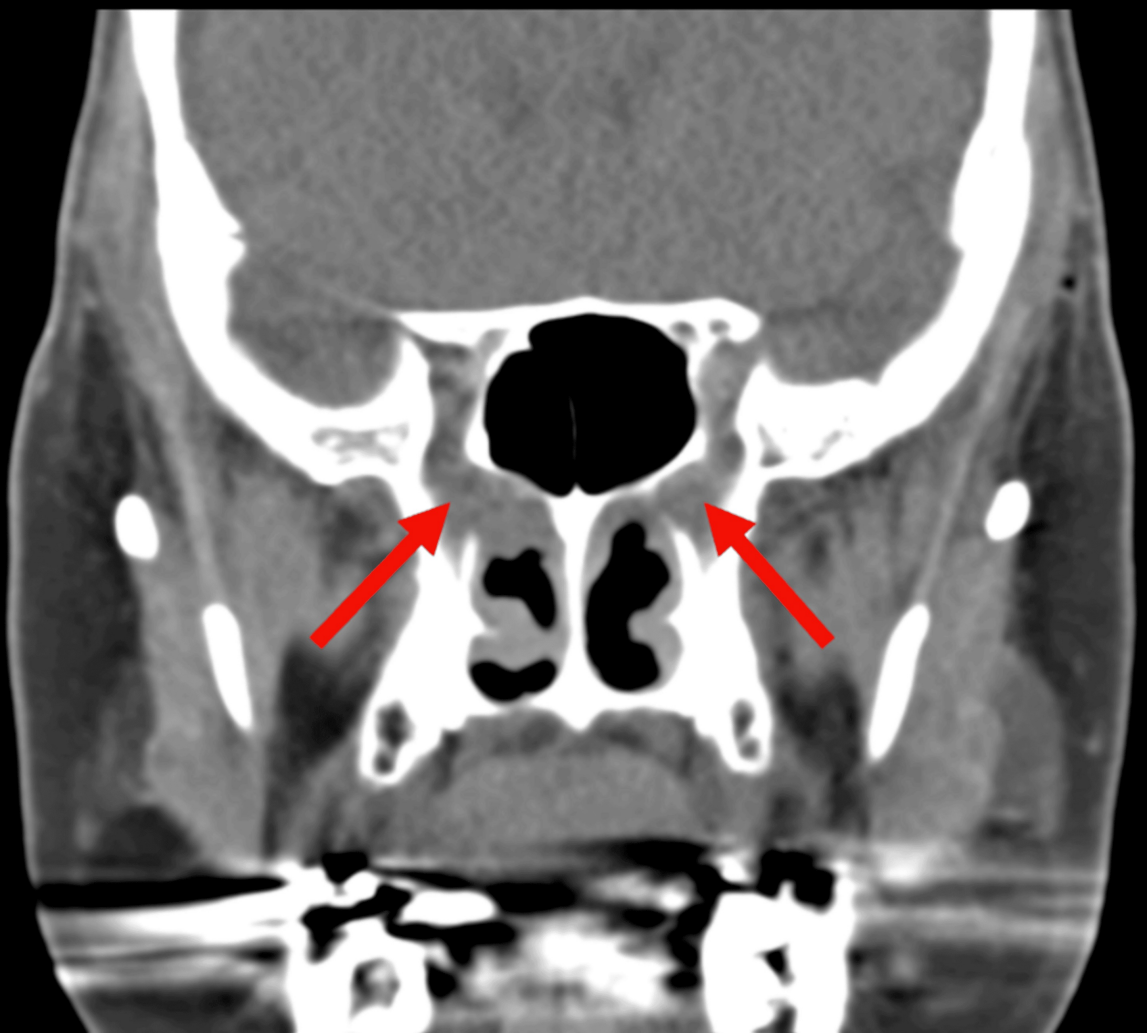
Coronal head CT with IV contrast demonstrating abnormal soft tissue thickening in the pterygopalatine fossa (arrows), extending into the sphenopalatine foramen, concerning for an infiltrative process. CT, Computed Tomography

Imaging studies, including MRI brain with and without contrast, were repeated to evaluate potential CNS involvement. The MRI showed enhancement of the right cranial nerve VII, as compared to the left, demonstrating an inflammatory process (Figure 1). A sinus CT further demonstrated abnormal soft tissue thickening in the right pterygopalatine fossa, extending into the sphenopalatine foramen. These findings were suggestive of an infiltrative or inflammatory process (Figure 2). Coronal imaging provided complementary visualization of the same lesion and extent of involvement (Figure 3). Laboratory testing revealed elevated inflammatory markers, including erythrocyte sedimentation rate (ESR) (29 mm/hr) and C-reactive protein (CRP) (10.0 mg/L), along with a positive cytoplasmic anti-neutrophil cytoplasmic antibody (c-ANCA) titer (1:640) and proteinase 3 (PR3) autoantibodies (60 U/mL). A lumbar puncture was conducted to rule out infectious or inflammatory causes. Opening pressure was not recorded at the time of the procedure. Cerebrospinal fluid (CSF) analysis showed 1 total nucleated cell/μL, normal protein at 42 mg/dL, and normal glucose at 64 mg/dL. Laboratory findings are summarized in Table 1. A biopsy of the pterygopalatine fossa soft tissue thickening was obtained, which demonstrated patchy neutrophilic inflammation, patchy eosinophilia, tissue necrosis with focally palisading histiocytes, and multinucleated giant cells consistent with granulomatous inflammation. Tissue culture demonstrated a few methicillin-resistant *Staphylococcus aureus* (MRSA). Collectively, these findings guided the diagnosis toward a vasculitic neuropathy secondary to GPA.

**Table 1:** Core laboratory and CSF findings with reference ranges. ANCA, Anti-neutrophil Cytoplasmic Antibody; ACE, Angiotensin-Converting Enzyme; CSF, Cerebrospinal Fluid; PMNs, Polymorphonuclear Leukocytes

Parameters	References	Patient Results
Erythrocyte Sedimentation Rate	0-18 (m/hr)	29
C-reactive Protein	< 8.0 (g/L)	10
ANCA Titer	< 1:20	1:640
ACE	0.00-2.5 (U/L)	0.8
Lysozyme	2.6-6.0 (mcg/mL)	5.3
Proteinase 3 Autoantibodies	< 2.0 (U/mL)	60
CSF Opening Pressure	6-20 (cm H_2_O)	Not recorded
CSF Total Nucleated Cells	1 /uL	≤ 5 /uL
CSF Protein	15-45 (mg/dL)	42
CSF Glucose	45-75 (mg/dL)	64
CSF Gram Stain	N/A	No PMNs, no organisms

Hospital course and management

Serum angiotensin-converting enzyme (ACE) was 0.8 U/L and lysozyme was 5.3 mcg/mL, both within normal reference ranges, making neurosarcoidosis less likely. These findings are summarized in Table 1.

**Figure 4:**
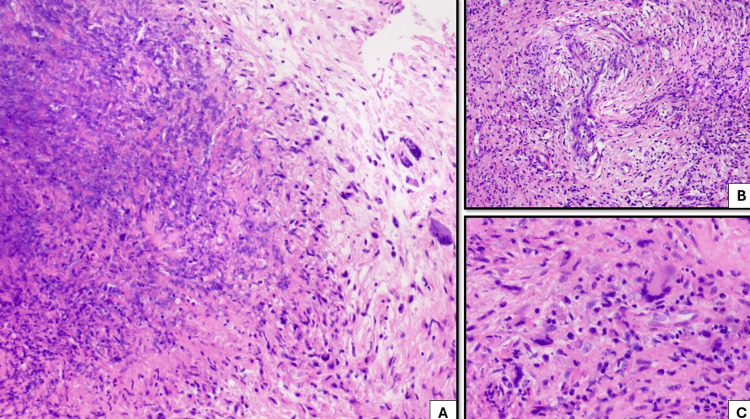
Marked inflammation with tissue necrosis and adjacent multinucleated giant cells (left, A, 200x, hematoxylin and eosin). Vasculitis with associated histiocytes, neutrophils, eosinophils, and multinucleated giant cells (top right, B, 200x, hematoxylin and eosin), better visualized at higher power (bottom right, C, 500x, hematoxylin and eosin). Combined, this constellation of findings is consistent with necrotizing granulomatous inflammation. Special stains for acid-fast bacilli and fungi are negative (not shown).

A biopsy of the pterygopalatine fossa soft tissue thickening revealed marked inflammation with areas of tissue necrosis and adjacent multinucleated giant cells. Histologic sections demonstrated vasculitis with associated histiocytes, neutrophils, eosinophils, and multinucleated giant cells. These findings were best visualized under hematoxylin and eosin staining at both intermediate (200x) and high (500x) magnification. The constellation of these histopathologic features is consistent with necrotizing granulomatous inflammation. Special stains for acid-fast bacilli and fungi were negative (Figure 4). Tissue culture demonstrated a few MRSA.

Autoimmune and paraneoplastic antibody panels were also obtained to rule out neuromuscular junction disorders, demyelinating neuropathies, and neoplastic syndromes. Anti-AChR blocking and modulating antibodies were 0%, and anti-GQ1b (Ganglioside Q1b) antibodies were 5 IV, all within negative ranges. Paraneoplastic antibodies, including ANNA-1 (Anti-neuronal Nuclear Antibody type 1), ANNA-2 (Anti-neuronal Nuclear Antibody type 2), PCA-1 (Purkinje Cell Antibody type 1), PCA-Tr (Purkinje Cell Antibody Tr (Truncated)), and CRMP-5-IgG (Collapsin Response Mediator Protein-5 Immunoglobulin G), were uniformly negative in CSF. These findings, summarized in Table 2, helped rule out conditions such as myasthenia gravis, Miller Fisher syndrome, and leptomeningeal carcinomatosis. Reference Table 2 for a complete autoimmune and paraneoplastic antibody panel, including anti-AChR, anti-GQ1b, and ANNA-1/2 results.

**Table 2:** Autoimmune and paraneoplastic antibody panel results. AChR, Acetylcholine Receptor; GQ1b, Ganglioside Q1b; ANNA-1 (Anti-Hu), Anti-neuronal Nuclear Antibody type 1 (Anti-Hu antibody); ANNA-2, Anti-neuronal Nuclear Antibody type 2; PCA-1, Purkinje Cell Antibody type 1; PCA-Tr, Purkinje Cell Antibody Tr (Truncated); CRMP-5-IgG, Collapsin Response Mediator Protein-5 Immunoglobulin G

Parameters	References	Patient Results
AChR Blocking Antibodies	0-26%	0%
AChR Modulating Antibodies	≤ 45%	0%
GQ1b Antibodies (IgG/IgM)	<30 IV (Negative)	5 IV (Negative)
ANNA-1 (Anti-Hu)	Negative	Negative
ANNA-2	Negative	Negative
PCA-1	Negative	Negative
PCA-Tr	Negative	Negative
CRMP-5-IgG	Negative	Negative
Paraneoplastic Interpretation	N/A	Negative

Urinalysis showed no proteinuria, hematuria, or casts, and serum creatinine levels remained stable between 0.65 and 0.88 mg/dL during the hospital course. Blood urea nitrogen (BUN) values ranged from 11 to 20 mg/dL, all within normal limits. These findings suggested preserved renal function, and no evidence of renal involvement was observed in this case of GPA. Collectively, these findings guided the diagnosis toward a vasculitic neuropathy secondary to GPA.

## Discussion

Cranial neuropathy in GPA is rare but well-documented in the literature. Involvement of multiple cranial nerves is often due to granulomatous inflammation and vasculitis affecting the cranial base [8]. This cranial nerve involvement likely reflects a dual mechanism of granulomatous inflammation at the skull base foramina and small-vessel vasculitis affecting the vasa nervorum, leading to both compressive and ischemic neuropathy. GPA has a predilection for ENT and skull base structures, which explains the frequency of lower cranial nerve involvement in some neurologic presentations of the disease [9,10]. The progression from unilateral to bilateral cranial nerve deficits in this patient suggests an aggressive disease course. The differential diagnoses included neuromuscular junction disorders such as myasthenia gravis, demyelinating syndromes such as Miller Fisher syndrome, inflammatory conditions like neurosarcoidosis, and neoplastic etiologies including leptomeningeal metastases. Anti-AChR antibodies and anti-GQ1b antibodies were negative, ruling out myasthenia gravis and Miller Fisher syndrome, respectively. The patient's pupil-sparing cranial nerve III palsy and lack of fluctuating or bulbar symptoms further supported the exclusion of myasthenia gravis on clinical grounds. Comprehensive CSF analysis revealed normal glucose, protein, and cell counts, with no pleocytosis or cytologic abnormalities, and no organisms or malignant cells identified.

Additionally, serum ACE and lysozyme levels were within normal limits, further arguing against neurosarcoidosis. Paraneoplastic antibody panels (e.g., ANNA-1/2 and Purkinje cell antibodies) and MRI imaging demonstrated no evidence of leptomeningeal enhancement or mass effect, effectively ruling out both neurosarcoidosis and leptomeningeal carcinomatosis. Skull base masses, such as lymphoma or invasive fungal sinusitis, were also considered. However, the absence of discrete mass-forming lesions on MRI, normal CSF profile, and biopsy showing necrotizing granulomatous inflammation were most consistent with GPA and not these other entities [10,11]. Ultimately, histopathologic confirmation of necrotizing granulomatous inflammation, along with a positive c-ANCA and PR3 profile, supported the diagnosis of GPA. PR3-ANCA is the most specific serologic marker for GPA, present in approximately 65%-75% of cases and particularly associated with ENT and neurologic manifestations. Its presence in this patient strongly reinforced the clinical diagnosis [12,13].

The variability in GPA presentation can often contribute to diagnostic delays. While classic manifestations of GPA include upper respiratory, renal, and pulmonary involvement, atypical presentations, such as isolated cranial neuropathy, can pose diagnostic challenges [14]. This highlights the importance of maintaining a broad differential and considering vasculitis in cases with progressive, unexplained neurological deficits. Fragoulis et al. conducted a retrospective study demonstrating the rarity of CNS involvement in GPA, further supporting the unusual nature of this case [5].

Hypertrophic cranial pachymeningitis is a rare but documented manifestation of GPA. While this case lacked direct pachymeningeal involvement, given the expanding literature, it remains an important diagnostic consideration. Söderström et al. [4] emphasized the complexity of CNS involvement in GPA, and De Luna et al. [3] further described how distinct clinical-radiological presentations of CNS disease in GPA can influence disease outcomes.

Furthermore, other cases have demonstrated a progressive course of GPA affecting cranial nerves, evolving from unilateral to bilateral involvement. Shil and Teir reported a case of bilateral facial palsy as a rare manifestation of GPA, highlighting how disease progression can mimic other neurological conditions [15]. Similarly, Söderström et al. described a rapidly progressive GPA case with cranial nerve involvement, initially presenting as unilateral hypoglossal nerve dysfunction before evolving into more extensive neuropathy [4]. These reports highlight the clinical variability and diagnostic complexity of GPA with cranial neuropathy.

Another feature in this case is the patient's response to immunosuppressive therapy. High-dose corticosteroids are fundamental in the initial management of GPA; however, in cases with significant neurologic involvement, the addition of rituximab, or cyclophosphamide, is often necessary to induce remission [1].

In this case, high-dose corticosteroids were initiated during hospitalization, and rituximab therapy was started approximately two and a half weeks after discharge, on February 6, following microbiological clearance and coordination with rheumatology. A second infusion was administered on February 20, consistent with the standard induction protocol. This timing aligns with clinical recommendations that emphasize resolving concurrent infections prior to initiating advanced immunosuppressive therapy.

Based on high-quality comparative studies, rituximab has demonstrated comparable or superior efficacy to cyclophosphamide in the treatment of GPA, particularly in relapsing cases or those associated with PR3-ANCA. Stone et al. found that rituximab was non-inferior to cyclophosphamide for induction therapy and more effective in relapsing disease [16]. More recently, Papuashvili et al. conducted a systematic review, indicating that rituximab may offer better remission rates and fewer long-term complications compared to cyclophosphamide, especially in terms of fertility preservation and infection risk [17].

Rituximab was selected in this case due to its favorable side effect profile compared to cyclophosphamide. It was initiated approximately 2.5 weeks after hospital discharge, following microbiological clearance and multidisciplinary coordination with rheumatology. The patient's prompt clinical improvement following rituximab initiation supports its effectiveness in severe, neurologically involved GPA. However, long-term follow-up is essential to monitor for relapses and potential complications of immunosuppression [18].

Lastly, while the multidisciplinary approach was crucial in coordinating timely and appropriate management, the diagnosis of GPA in this case was substantiated not only through clinical manifestations but also by key laboratory findings that are consistent with reported cases of CNS-involved GPA. These included a markedly positive c-ANCA (1:640), elevated PR3 (60 U/mL), and normal CSF parameters without pleocytosis or cytologic abnormalities - findings which align with prior descriptions of GPA with neurologic presentations. Negative serum ACE and lysozyme levels also helped exclude alternative inflammatory etiologies, such as neurosarcoidosis. Histopathologic evaluation of the pterygopalatine lesion demonstrated necrotizing granulomatous inflammation with tissue necrosis, palisading histiocytes, eosinophils, neutrophils, and multinucleated giant cells - hallmarks of GPA. Special stains for fungi and acid-fast bacilli were negative, helping rule out infectious mimickers. Together, these results, in conjunction with biopsy-proven necrotizing granulomatous inflammation, confirmed the diagnosis. Collaboration between rheumatology, neurology, ophthalmology, and infectious disease specialists ensured a thorough evaluation and timely initiation of targeted therapy [19]. This emphasizes the importance of both analytical rigor and an integrated, team-based approach in managing diagnostically challenging autoimmune conditions such as GPA.

## Conclusions

This case highlights the importance of maintaining a high index of suspicion on the differential for GPA in patients presenting with progressive cranial nerve deficits, even in the absence of prototypical systemic features. The transition from unilateral to bilateral involvement emphasized the disease’s aggressive nature and diagnostic complexity. Early diagnosis and initiation of immunosuppressive therapy, including rituximab, were critical in achieving clinical improvement and preventing long-term complications. Ultimately, collaboration through a multidisciplinary approach remains crucial in navigating such rare and diagnostically challenging presentations of GPA.

## References

[1] Iannella G, Greco A, Granata G (2016). Granulomatosis with polyangiitis and facial palsy: literature review and insight in the autoimmune pathogenesis. Autoimmun Rev.

[2] Peters JE, Gupta V, Saeed IT, Offiah C, Jawad AS (2018). Severe localised granulomatosis with polyangiitis (Wegener's granulomatosis) manifesting with extensive cranial nerve palsies and cranial diabetes insipidus: a case report and literature review. BMC Neurol.

[3] De Luna G, Terrier B, Kaminsky P (2015). Central nervous system involvement of granulomatosis with polyangiitis: clinical-radiological presentation distinguishes different outcomes. Rheumatology (Oxford).

[4] Söderström A, Revaz S, Dudler J (2015). Cranial neuropathies in granulomatosis with polyangiitis (Wegener's): a case-based review. Clin Rheumatol.

[5] Fragoulis GE, Lionaki S, Venetsanopoulou A, Vlachoyiannopoulos PG, Moutsopoulos HM, Tzioufas AG (2018). Central nervous system involvement in patients with granulomatosis with polyangiitis: a single-center retrospective study. Clin Rheumatol.

[6] Jeong SM, Park JH, Lee JI, Nam KE, Lee JS, Kim JH (2016). Progressive bilateral facial palsy as a manifestation of granulomatosis with polyangiitis: a case report. Ann Rehabil Med.

[7] Watts RA, Carruthers DM, Scott DG (1995). Epidemiology of systemic vasculitis: changing incidence or definition?. Semin Arthritis Rheum.

[8] Kiessling PT, Marinelli JP, Peters PA, DeLone DR, Lane JI, Koster MJ, Carlson ML (2020). Cranial base manifestations of granulomatosis with polyangiitis. Otolaryngol Head Neck Surg.

[9] Potentas-Policewicz M, Fijolek J (2024). Granulomatosis with polyangiitis: clinical characteristics and updates in diagnosis. Front Med (Lausanne).

[10] Qureshi HA, Bandhlish A, DeConde RP, Humphreys IM, Abuzeid WM, Jafari A (2022). Initial presentation of granulomatosis with polyangiitis as progressive skull base osteomyelitis. ORL J Otorhinolaryngol Relat Spec.

[11] Greco A, Marinelli C, Fusconi M (2016). Clinic manifestations in granulomatosis with polyangiitis. Int J Immunopathol Pharmacol.

[12] Martinez Del Pero M, McKiernan D, Jani P (2014). Presentation and initial assessment of ENT problems in patients with granulomatosis with polyangiitis (Wegener's). J Laryngol Otol.

[13] Pagnoux C, Villa-Forte A (2023). Granulomatosis with polyangiitis. Orphan Lung Diseases.

[14] Marini K, Garefis K, Skliris JP (2024). Granulomatosis with polyangiitis: multiple cranial nerve manifestations and nasopharyngeal pseudotumor. Ear Nose Throat J.

[15] Shil RS, Teir JA (2021). An interesting case of bilateral facial palsy due to granulomatosis with polyangiitis. Case Rep Rheumatol.

[16] Stone JH, Merkel PA, Spiera R (2010). Rituximab versus cyclophosphamide for ANCA-associated vasculitis. N Engl J Med.

[17] Papuashvili P, Vepkhishvili G, Makaridze T, Popiashvili G (2024). Impact of rituximab on remission rates in granulomatosis with polyangiitis: a systematic review. Cureus.

[18] Miłkowska-Dymanowska J, Laskowska P (2019). Untypical manifestations of granulomatosis with polyangiitis—a review of the literature. SN Compr Clin Med.

[19] Kim SH, Park J, Bae JH, Cho MS, Park KD, Jeong JH (2013). ANCA-negative Wegener's granulomatosis with multiple lower cranial nerve palsies. J Korean Med Sci.

